# Esters of the Marine-Derived Triterpene Sipholenol A Reverse P-GP-Mediated Drug Resistance

**DOI:** 10.3390/md13042267

**Published:** 2015-04-14

**Authors:** Yongchao Zhang, Yun-Kai Zhang, Yi-Jun Wang, Saurabh G. Vispute, Sandeep Jain, Yangmin Chen, Jessalyn Li, Diaa T. A. Youssef, Khalid A. El Sayed, Zhe-Sheng Chen

**Affiliations:** 1 Department of General Surgery, The Affiliated Cancer Hospital of Zhengzhou University/Henan Cancer Hospital, Zhengzhou 450003, Henan, China; E-Mail: zhangycqh@yahoo.com; 2 Department of Pharmaceutical Sciences, College of Pharmacy and Health Sciences, St. John’s University, Queens, NY 11439, USA; E-Mails: helloyunyun@live.com (Y.-K.Z.); yijun.wang11@my.stjohns.edu (Y.-J.W.); sgvispute@gmail.com (S.G.V.); jessblue67@gmail.com (J.L.); 3 Department of Basic Pharmaceutical Sciences, School of Pharmacy, University of Louisiana at Monroe, 1800 Bienville Drive, Monroe, LA 71201, USA; E-Mails: sandeep1602@gmail.com (S.J.); elsayed@ulm.edu (K.A.E.S.); 4 Ernest Mario School of Pharmacy, Rutgers, the State University of New Jersey, New Brunswick, NJ 08901, USA; E-Mail: m.and.m.rox.my.sox@gmail.com; 5 Department of Natural Products, Faculty of Pharmacy, King Abdulaziz University, 21589 Jeddah, Saudi Arabia; E-Mail: dyoussef@kau.edu.sa

**Keywords:** sipholenol A, ABC transporter, multidrug resistance, P-gp/ABCB1, BCRP/ABCG2, MRP1/ABCC1, marine natural products

## Abstract

Our previous studies showed that several sipholane triterpenes, sipholenol A, sipholenone E, sipholenol L and siphonellinol D, have potent reversal effect for multidrug resistance (MDR) in cancer cells that overexpressed P-glycoprotein (P-gp/ABCB1). Through comparison of cytotoxicity towards sensitive and multi-drug resistant cell lines, we identified that the semisynthetic esters sipholenol A-4-*O*-acetate and sipholenol A-4-*O*-isonicotinate potently reversed P-gp-mediated MDR but had no effect on MRP1/ABCC1 and BCRP/ABCG2-mediated MDR. The results from [^3^H]-paclitaxel accumulation and efflux studies suggested that these two triterpenoids were able to increase the intracellular accumulation of paclitaxel by inhibiting its active efflux. In addition, western blot analysis revealed that these two compounds did not alter the expression levels of P-gp when treated up to 72 h. These sipholenol derivatives also stimulated the ATPase activity of P-gp membranes, which suggested that they might be substrates of P-gp. Moreover, *in silico* molecular docking studies revealed the virtual binding modes of these two compounds into human homology model of P-gp. In conclusion, sipholenol A-4-*O*-acetate and sipholenol A-4-*O*-isonicotinate efficiently inhibit the P-gp and may represent potential reversal agents for the treatment of multidrug resistant cancers.

## 1. Introduction

Multi-drug Resistance (MDR) is a major clinical obstacle in the treatment of cancer. The cause of MDR is diversely characterized by multiple alterations in protein expression. The mechanism of drug resistance can occur due to increased drug efflux, reduced drug uptake, activation of detoxifying proteins, activation of DNA repair, and disruption of apoptotic signaling pathways [1,2,3]. Among these, increased drug extrusion mediated by P-glycoprotein (P-gp/ABCB1) is a major factor [4]. P-gp is a 170-kD transmembrane transporter in the family of ATP-binding cassette (ABC) transporters that are distributed widely across various organs [5,6]. P-gp facilitates the efflux of cytotoxic compounds out of cells with the energy generated by ATP hydrolysis [7]. The transporter also recognizes a large number of therapeutic drugs and is overexpressed in cancer cells [8]. This significantly reduces the therapeutic efficacy of chemotherapeutic agents and disposes the patient to subpar treatments.

Many scientists aim to inhibit the P-gp transporter to reverse MDR and again re-sensitize the cancer cells at clinically effective doses of chemotherapeutic agents. Tsuruo *et al.* in 1981 discovered that verapamil could effectively reverse P-gp-mediated drug resistance *in vitro*, opening the doors to a cascade of compounds to be evaluated for this ability [9]. The first generation agents that can inhibit P-gp-mediated MDR are currently US Food and Drug Administration (FDA) approved therapeutic compounds such as verapamil, quinidine, amiodarone, tamoxifen, progesterone, and cyclosporin A [10]. Although it is attractive to utilize these agents to treat cancer patients with indicated co-morbidities, research has shown that these compounds are effective as MDR reversal agents either at much higher doses or may relate to various undesirable side effects. For example, tamoxifen exhibited 50% of P-gp mediated drug accumulation at 60 μM [11], and cyclosporin A treatment may be associated with potentially serious adverse drug reactions. Thus, second-generation agents with higher affinity for the P-gp transporter were developed with an aim to improve MDR inhibition. Second-generation inhibitors such as PSC-833 (a non-immunosuppressive analogue of cyclosporin A) also failed in clinical trials due to unwarranted pharmacokinetic interactions [2,12]. To minimize these drug interactions, third generation of MDR inhibitors are currently being designed to modulate P-gp activity at nanomolar concentrations, to increase the potency and decrease adverse drug reactions [13].

Although, exploring the marine environment for new therapeutic compounds is not a recent strategy, the amount of unknown correlates to the potential it holds in the ecosystem. Marine environment houses a plethora of bioactive compounds that express diverse therapeutic effects, such as being anti-inflammatory, antiretroviral, analgesic, antitumor, and immunomodulator [14]. In the past, extensive screening of marine sponge compounds has led to the discovery of many potential MDR-reversal agents [15,16]. A group of compounds, the sipholane triterpenoids from the Red Sea sponge *Siphonochalina siphonella*, potently reversed P-gp-mediated MDR in cancer cells. Previous research showed that sodwanones from the Indian Ocean sponge *Axinella weltneri* demonstrated anticancer properties [17,18]. Due to the structural similarity of sodwanes to polyepoxysqualene-derived triterpenoids, it was hypothesized that sipholane triterpenoids could also exhibit potential anticancer effects. 

Sipholane triterpenes were extracted from *Callyspongia siphonella*, a Red Sea marine sponge. Several semisynthetic analogs of sipholenol A were synthesized and showed potent breast cancer migration inhibitory activities [19,20]. These sipholenol analogs have close structural and pharmacophoric similarities to the previously described compounds that showed drug-resistance reversal properties. They were first tested for cytotoxicity on cancer cells and then for reversal of P-gp mediated MDR. Among these compounds, sipholenol A-4-*O*-acetate (SSJ26) and sipholenol A-4-*O*-isonicotinate (SSJ32) demonstrated P-gp inhibition comparable to that of verapamil. In this study, these two compounds were further analyzed to elucidate their mechanism of action and strengthen their value as potential P-gp-mediated drug resistance reversal agents. 

## 2. Materials and Methods

### 2.1. Materials

**Figure 1:**
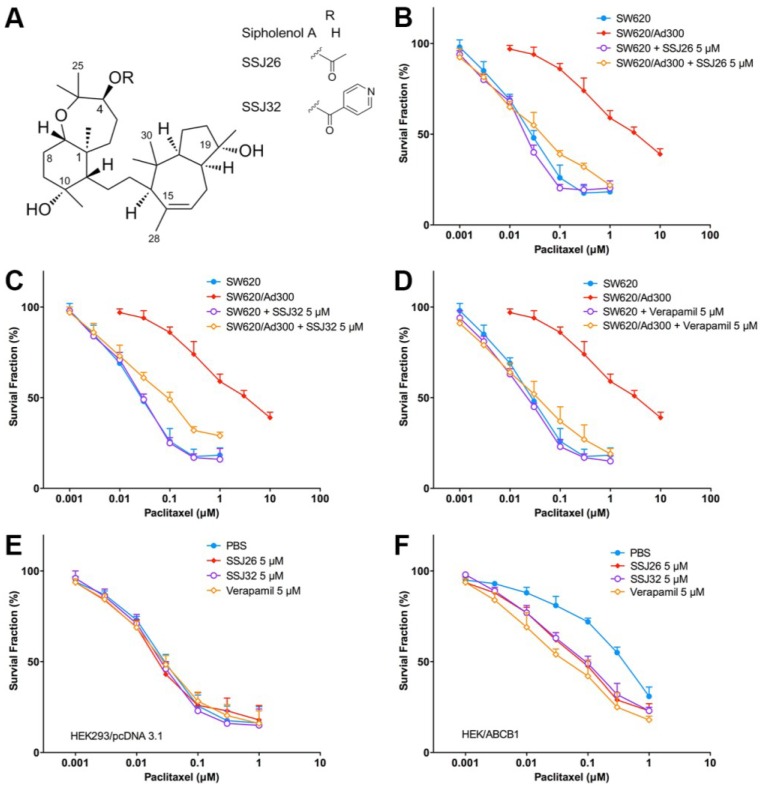
Reversal effect of SSJ26 and SSJ32 against P-gp-mediated MDR in representative cell survival curves. (**A**) Chemical structures of sipholenol A, SSJ26 and SSJ32; (**B**) Concentration-survival curves of SW620 and SW620/Ad300 cell lines treated with combination of SSJ26 and paclitaxel; (**C**) Concentration-survival curves of SW620 and SW620/Ad300 cell lines treated with combination of SSJ32 and paclitaxel; (**D**) Concentration-survival curves of SW620 and SW620/Ad300 cell lines treated with combination of verapamil and paclitaxel; (**E**) Concentration-survival curves of HEK293/pcDNA3.1 cell lines treated with SSJ26, SSJ32 or verapamil in combination of paclitaxel; (**F**) Concentration-survival curves of HEK/ABCB1 cell lines treated with SSJ26, SSJ32 or verapamil in combination of paclitaxel. Cell survival rate was determined by the MTT assay as described in Section 2. Data points with error bars represent the mean ± SD. Each above figure is a representative of three independent experiments, each done in triplicate.

[^3^H]-paclitaxel was purchased from Moravek Biochemicals, Inc. (Brea, CA, USA). Monoclonal antibody C-219 (against P-gp) and loading control antibody BA3R (against β-actin) were purchased from Thermo Fisher Scientific (Rockford, IL, USA). Sipholane analogs were previously synthesized and the structures are shown in Figure 1A and Supplementary Information Figure S1. Fumitremorgin C (FTC) was synthesized by Thomas McCloud Developmental Therapeutics Program, Natural Products Extraction Laboratory, NCI, NIH (Bethesda, MD, USA) and it was a gift from Susan E Bates and Robert W. Robey. The Gentest ATPase kit was purchased from BD Biosciences (San Jose, CA, USA). Doxorubicin, paclitaxel, vincristine, verapamil, 3-(4,5-dimethylthiazol-2-yl)-2,5-diphenyltetrazolium bromide (MTT) and other chemicals were purchased from Sigma Chemical Co. (St. Louis, MO, USA). PAK-104P was a gift of Shin-Ichi Akiyama (Kagoshima University, Kagoshima, Japan) from Nissan Chemical Ind. Co., Ltd. (Chiba, Japan).

### 2.2. Cell lines and Cell Culture

HEK293/pcDNA3.1, HEK/ABCB1, HEK/ABCG2 and HEK/ABCC1 cells were generated by transfecting the HEK293 cells with either empty pcDNA3.1 vector, *ABCB1* expression vector, *ABCG2* expression vector or *ABCC1* expression vector respectively [21]. HEK/ABCC1 was generated in the laboratory of Suresh V. Ambudkar (NCI, NIH, Bethesda, MD, USA). The human colon cancer cell line SW620 and doxorubicin-selected P-gp overexpressing SW620/Ad300 cell line were used for the reversal study. SW620, SW620/Ad300 and HEK/ABCG2 cells were also kindly provided by Susan Bates and Robert Robey (NCI, NIH, Bethesda, MD, USA). All the cell lines were grown as adherent monolayers in flasks with DMEM culture medium (GE Healthcare Life Sciences, Logan, UT, USA) supplemented with 10% bovine serum in a humidified incubator containing of 5% CO_2_ at 37 °C.

### 2.3. Cell Cytotoxicity by MTT Assay

The MTT colorimetric assay was used to detect the sensitivity of cells to anticancer drugs. Cells were harvested with trypsin treatment. After washing with PBS, cells were resuspended in the culture media. Cells with a final concentration of 5 × 10^3^ cells/well were seeded evenly into 96-well plates with 160 μL media. For the reversal experiments, SSJ compounds, verapamil, FTC or PAK104P (20 μL/well) was added followed by different concentrations of chemotherapeutic drugs (20 μL/well) into designated wells. After 72 h of incubation, 20 μL of MTT solution (5 mg/mL) was added to each well, and the plate was further incubated for 4 h, allowing viable cells to convert the yellow colored MTT into dark-blue formazan crystals. Subsequently, the medium was discarded, and 100 μL of dimethylsulfoxide (DMSO) was added into each well to dissolve the formazan crystals. The absorbance was determined at 570 nm by an OPSYS Microplate Reader from DYNEX Technologies Inc. (Chantilly, VA, USA) followed previously described protocol [22]. The fold of resistance was calculated by dividing the IC_50_ (concentrations required to inhibit growth by 50%) of the MDR cells by that of the parental sensitive cells.

### 2.4. Preparation of Total Cell Lysates

SW620/Ad300 cells were incubated with SSJ26 and SSJ32 at 5 μM for different time periods (0, 24, 48, and 72 h). Total cell lysates were prepared by harvesting the cells and rinsing twice with ice cold PBS, then by incubating cells for 30 min on ice with radioimmunoprecipitation assay (RIPA) buffer (1 × PBS, 0.1% SDS, 1% Nonidet P-40, 0.5% sodium deoxycholate, 10 mg/mL leupeptin, 100 mg/mL p-aminophenylmethylsulfonyl fluoride and 10 mg/mL aprotinin) followed by centrifugation at 12,000× *g* at 4 °C for 15 min. The supernatant containing total cell lysates was stored at −80 °C until needed for experiments. The protein concentration was determined by bicinchoninic acid (BCA™)-based protein assay (Thermo Scientific, Rockford, IL, USA).

### 2.5. Western Blot Analysis

Equal amounts of total cell lysates (50 μg protein) were resolved by sodium dodecyl sulfate polycrylamide gel electrophoresis (SDS-PAGE) and electrophoretically transferred onto polyvinylidene fluoride (PVDF) membranes. After incubation in a blocking solution in TBST buffer (10 mM Tris-HCl, pH 8.0, 150 mM NaCl, and 0.1% Tween 20) for 1 h at room temperature, the membranes were immunoblotted overnight with primary monoclonal antibody against P-gp and against β-actin at 4 °C, and were then incubated for 4 h at room temperature with horseradish peroxide (HRP)-conjugated secondary antibody (1:2000 dilution) modified from our previous protocols [23]. The protein-antibody complex was detected by enhanced chemiluminescence detection system (Amersham, NJ, USA).

### 2.6. [^3^H]-Paclitaxel Accumulation and Efflux Assays

The effect of SSJ compounds on the intracellular accumulation of paclitaxel in SW620 and SW620/Ad300 cells was determined by measuring the intracellular accumulation of [^3^H]-paclitaxel in these cells. Cells were seeded in triplicate at 3 × 10^5^ cells/well into 6-well plates. The next day, the cells were pre-incubated with or without the reversal compound for 2 h at 37 °C. Intracellular drug accumulation was measured by incubating cells with 0.01 μM [^3^H]-paclitaxel for 2 h in the presence or absence of the inhibitor at 37 °C. The cells were washed three times with ice-cold PBS, trypsinized and lysed in 10 mM lysis buffer (pH 7.4, containing 1% Triton X-100 and 0.2% SDS). An aliquot of cells was used to analyze cell number, and the remaining cells were pelleted at 4°C and washed three more times with ice-cold PBS. Each sample was placed in scintillation fluid and radioactivity was measured by a liquid scintillation counter following our previous protocol [24].

In the efflux study, cells were incubated with 0.1 μM [^3^H]-paclitaxel as the same in the accumulation study. After washing three times with cold PBS, the cells were incubated at 37 °C in fresh medium in the presence or absence of 5 μM SSJ26, or 5 μM SSJ32. After 0, 30, 60, and 120 min, the cells were washed three times with ice-cold PBS, trypsinized and lysed in 10 mM lysis buffer (pH 7.4, containing 1% Triton X-100 and 0.2% SDS). The cells were then processed for measurement of radioactivity by Packard TRI-CARB 1900CA liquid scintillation analyzer as previously described [25].

### 2.7. ATPase Assay of SSJ26 and SSJ32

The P-gp associated ATPase activity was measured by BD Gentest ATPase kit. The assay was carried out in white opaque 96-well multiplates in triplicate. Recombinant human P-gp membrane (5 mg/mL) was quickly thawed and diluted to 1 mg/mL with assay buffer. Sodium orthovanadate (Na_3_VO_4_) was used as an ATPase inhibitor. Various concentrations of SSJ26 and SSJ32 were incubated in 20 μg (20 μL) diluted recombinant human P-gp membrane at 37 °C for 5 min. The reaction is initiated by adding 20 mL of 15 mM Mg^2+^ ATP to all wells. At this point, each P-gp reaction contains 5 mM ATP. The plate was incubated at 37 °C for 40 min with brief mixing using a plate shaker. Luminescence initiated by ATP detection buffer. After incubation at 37 °C for 20 min to allow luminescent signal to develop, the untreated white opaque 96-well multiplate was read on luminometer (SpectraMax M5, molecular devices, Sunnyvale, CA, USA). The changes of relative light units (DRLU) were determined by comparing Na_3_VO_4_-treated samples with SSJ26 and SSJ32 combination-treated groups [26].

### 2.8. Molecular Docking Analysis

SSJ26 and SSJ32 were built and prepared as ligands by our previous molecular modeling protocols [27]. The output files containing at most 100 unique conformers of SSJ26 or SSJ32 were used as input for docking simulations into human homology P-gp.

Human P-gp homology model based on mouse Abcb1 was kindly provided by S. Aller and was used to generate grid for docking [28]. Schrödinger Suite 2014-4 Protein Preparation Wizard (Epik version 3.0; Impact version 6.5, Schrödinger, LLC, New York, NY, USA, 2014) protocol was followed for protein preparation and refinement. Conserved residues previously identified as interacting with cyclic peptides and drugs were selected as centroid for generating docking grid. Docking grid was refined as an enclosing box with length of 20 Å by Glide version 6.4 (Schrödinger, LLC, New York, NY, USA, 2014).

Docking of SSJ26 or SSJ32 into human P-gp was performed using the “Extra Precision” (XP) mode of Glide version 6.4 (Schrödinger, LLC, New York, NY, USA, 2014). The value of Glide Emodel was ranked to determine the best-docked pose for SSJ26 or SSJ32 [29]. The two selected docked poses were used for graphic analysis. XP Glidescores calculated by Glide version 6.4 (Schrödinger, LLC, New York, NY, USA, 2014) were used to rank these two poses. All computations were carried out on a Dell Precision 490n dual processor with Linux OS (Ubuntu 12.04 LTS, Canonical Group Limited, London, UK).

### 2.9. Statistical Analysis

All experiments were performed as triplicates and the differences were determined by using the Student’s *t*-test. The statistical significance was determined at *p* < 0.05 and *p* < 0.01.

## 3. Results 

### 3.1. Screening for Potential Inhibitors of P-GP Transporter in Comparison with Verapamil

Sipholane analogs (14 compounds) were first tested for cytotoxicity using MTT assay on colon cancer cells SW620 (drug-sensitive) and SW620/Ad300 (drug-resistant). The IC_50_ values of all compounds were above 30 μM (Supplementary Information Table S1). Because these compounds were used at a concentration of 5 μM, which is the non-toxic concentration for all of them, they were considered to have no direct cytotoxic effect on cells.

Sipholane analogs were then screened for P-gp inhibitory effects in comparison to verapamil. Fold-of-resistance (FR) was calculated to determine which compounds could effectively increase the sensitivity of drug-resistant cells to chemotherapeutic agent. Cell viability was recorded using MTT assay after the following treatments were applied to the same cell lines. Doxorubicin, a known substrate of P-gp transporter, was used as baseline factor. Verapamil was used as positive control inhibitor of P-gp. SSJ26 and SSJ32 were found to be comparable to verapamil in reversing P-gp-mediated drug resistance as shown in Table 1; therefore, these two compounds were selected for further tests.

**Table 1:** Effect of 14 sipholane analogs on inhibition of P-gp mediated resistance to doxorubicin in SW620 and SW620/Ad300.

Treatment	SW620		SW620/Ad300	
	IC_50_ ± SD ^a^ (μM)	FR ^b^	IC_50_ ± SD ^a^ (μM)	FR ^b^
Doxorubicin	0.0196 ± 0.0022	1.00	4.1082 ± 0.4112	209.60
+Verapamil (5 μM)	0.0167 ± 0.0027	0.85	0.0876 ± 0.0015	4.47 **
+SSJ20 (5 μM)	0.0189 ± 0.0019	0.96	4.2660 ± 0.4016	217.65
+SSJ25 (5 μM)	0.0194 ± 0.0018	0.99	4.0939 ± 0.0083	208.87
+SSJ26 (5 μM)	0.0176 ± 0.0017	0.90	0.0941 ± 0.5238	4.80 **
+SSJ27 (5 μM)	0.0169 ± 0.0019	0.86	3.4125 ± 0.3317	174.11 *
+SSJ28 (5 μM)	0.0202 ± 0.0012	1.03	4.2550 ± 0.3567	217.09
+SSJ29 (5 μM)	0.0195 ± 0.0018	1.00	3.1563 ± 0.4173	161.04 *
+SSJ30 (5 μM)	0.0190 ± 0.0021	0.97	4.2027 ± 0.3513	214.42
+SSJ31 (5 μM)	0.0186 ± 0.0016	0.95	4.2814 ± 0.5122	218.44
+SSJ32 (5 μM)	0.0188 ± 0.0019	0.96	0.1312 ± 0.0101	6.69 **
+SSJ33 (5 μM)	0.0195 ± 0.0020	0.99	2.9758 ± 0.2593	151.83 *
+SSJ34 (5 μM)	0.0218 ± 0.0019	1.11	4.2762 ± 0.4135	218.17
+SSJ35 (5 μM)	0.0231 ± 0.0023	1.18	4.3574 ± 0.5143	222.32
+SSJ36 (5 μM)	0.0169 ± 0.0018	0.86	3.4676 ± 0.4491	176.92
+SSJ37 (5 μM)	0.0167 ± 0.0015	0.85	3.9948 ± 0.4798	203.82

^a^ Cell survival was determined by MTT assay as described in Section 2. Data are represented as mean ± SD of at least three independent experiments performed in triplicate; ^b^ Fold-of-resistance (FR) was determined by dividing the IC_50_ value for doxorubicin of SW620/Ad300 with or without reversal agent, or of SW620 in the presence of reversal agent by the IC_50_ value for doxorubicin of SW620 in the absence of reversal agent; Unpaired Student’s t-test was employed to analyze the differences between FR values. * *p* < 0.05; ** *p* < 0.01.

### 3.2. Effect of SSJ26 and SSJ32 in P-GP Overexpressing Cancer and ABCB1-Transfected Cells

Based on the previous experiment, SSJ26 and SSJ32 were chosen and studied further with three different concentrations. Doxorubicin was applied to SW620 and SW620/Ad300 alone and in combination with various concentrations of the potential P-gp modulators: SSJ26 and SSJ32. As shown in Table 2, the complete reversal of P-gp-mediated resistance is defined as FR = 1.00. When the SW620/Ad300 (drug-resistant) cells were treated with doxorubicin, it required 209-times the amount of drug needed to achieve the same IC_50_ as that in SW620 (drug-sensitive) cells. This resistance could be significantly reduced by the addition of 5 μM of verapamil, decreasing the fold-of-resistance (FR) from 209 to 4.47. Out of the fourteen compounds, SSJ26 and SSJ32 achieved the similar effect with FR reduced to 4.80 and 6.69, respectively. The fold-of-resistance decreased as concentrations of SSJ26 and SSJ32 increased, demonstrating that the reversal occurs in a concentration-dependent manner. Another chemotherapeutic drug and P-gp substrate, paclitaxel, was also used to test the reversal effect of these compounds. Similar to doxorubicin, SSJ26 and SSJ32 improved paclitaxel sensitivity in P-gp overexpressing SW620/Ad300 cells. Finally, cisplatin, a chemotherapeutic agent that is not a P-gp substrate, was applied with the 5 μM of verapamil, SSJ26, or SSJ32. Since cisplatin is not susceptible to resistance by P-gp [7], the resistant fold of SW620/Ad300 as a ratio to the IC_50_ of SW620 remained at around 1.00 as predicted. Similarly, FR values did not differ significantly with the treatment of verapamil, SSJ26 or SSJ32. This strengthens the hypothesis that SSJ26 and SSJ32 reverse P-gp-mediated resistance in a concentration-dependent manner.

**Table 2:** SSJ26 and SSJ32 reverse the P-gp-mediated drug resistance to doxorubicin and paclitaxel.

Treatment	SW620		SW620/Ad300	
	IC_50_ ± SD ^a^ (μM)	FR ^b^	IC_50_ ± SD ^a^ (μM)	FR ^b^
Doxorubicin	0.0201 ± 0.0023	(1.00)	4.3221 ± 0.4513	(215.16)
+SSJ26 (1.25 μM)	0.0199 ± 0.0018	(0.99)	0.5314 ± 0.05134	(26.44) *
+SSJ26 (2.5 μM)	0.0185 ± 0.0017	(0.92)	0.3212 ± 0.09021	(15.98) *
+SSJ26 (5 μM)	0.0181 ± 0.0021	(0.90)	0.1017 ± 0.01022	(5.06) **
+SSJ32 (1.25 μM)	0.0210 ± 0.0025	(1.05)	0.6354 ± 0.0724	(31.61) *
+SSJ32 (2.5 μM)	0.0199 ± 0.0021	(0.99)	0.3317 ± 0.0413	(16.50) *
+SSJ32 (5 μM)	0.0189 ± 0.0017	(0.94)	0.1528 ± 0.0132	(7.60) **
+Verapamil (5 μM)	0.0179 ± 0.0015	(0.89)	0.0923 ± 0.0058	(4.59) **
Paclitaxel	0.0072 ± 0.0010	(1.00)	2.7402 ± 0.2813	(380.58)
+SSJ26 (1.25 μM)	0.0063 ± 0.0008	(0.88)	0.2046 ± 0.0194	(28.42) *
+SSJ26 (2.5 μM)	0.0063 ± 0.0009	(0.92)	0.1081 ± 0.0098	(15.01) *
+SSJ26 (5 μM)	0.0057 ± 0.0006	(0.79) *	0.0737 ± 0.0084	(10.23) **
+SSJ32 (1.25 μM)	0.0068 ± 0.0005	(0.94)	0.2977 ± 0.0313	(41.35) *
+SSJ32 (2.5 μM)	0.0064 ± 0.0006	(0.89)	0.1110 ± 0.0092	(15.41) *
+SSJ32 (5 μM)	0.0061 ± 0.0005	(0.85)	0.0872 ± 0.0095	(12.12) **
+Verapamil (5 μM)	0.0062 ± 0.0007	(0.86)	0.0658 ± 0.0068	(9.14) **
Cisplatin	1.8203 ± 0.1744	(1.00)	2.3217 ± 0.3353	(1.28)
+SSJ26 (5 μM)	1.8556 ± 0.2919	(1.02)	2.1842 ± 0.3148	(1.20)
+SSJ32 (5 μM)	1.7453 ± 0.2434	(0.96)	2.1772 ± 0.0210	(1.19)
+Verapamil (5 μM)	1.8012 ± 0.2017	(0.99)	2.0313 ± 0.0251	(1.12)

^a^ IC_50_ values are represented as mean ± SD of at least three independent experiments performed in triplicate; ^b^ Values represent the fold-of-resistance (FR) obtained by dividing IC_50_ value of antineoplastic drug in SW620 and SW620/Ad300 cells with or without reversal agent divided by the IC_50_ value of respective antineoplastic drug in SW620 cells without reversal agent. Cell survival assay was determined by the MTT assay as described in Section 2. Verapamil was used as a positive control of P-gp inhibitor; * *p* < 0.05; ** *p* < 0.01 *versus* the control group without reversal agent.

Drug selected SW620/Ad300 cells may have multiple drug resistant mechanisms besides P-gp-mediated action. Therefore, the effect of sipholane analogs on P-gp was studied in HEK293 cells transfected with the *ABCB1* gene expression vector or empty pcDNA3.1 vector. The transfected HEK/ABCB1 cell was developed to solely exhibit P-gp-mediated resistance. In this cell line, the resistant folds were more significantly reduced by verapamil, SSJ26, and SSJ32 as shown in Table 3. These findings further support the hypothesis that SSJ26 and SSJ32 mainly affect P-gp.

**Table 3:** SSJ26 and SSJ32 reverse the P-gp-mediated drug resistance to paclitaxel in HEK293/pcDNA3.1 and HEK/ABCB1 cells.

Treatment	HEK293/pcDNA3.1		HEK/ABCB1	
	IC_50_ ± SD ^a^ (μM)	FR ^b^	IC_50_ ± SD ^a^ (μM)	FR ^b^
Paclitaxel	0.0224 ± 0.0030	(1.00)	0.9912 ± 0.1504	(44.25)
+SSJ26 (1.25 μM)	0.0279 ± 0.0047	(1.25)	0.1980 ± 0.0586	(8.84) **
+SSJ26 (5 μM)	0.0242 ± 0.0019	(1.08)	0.0389 ± 0.0040	(1.74) **
+SSJ32 (1.25 μM)	0.0254 ± 0.0025	(1.13)	0.2824 ± 0.016	(12.61) *
+SSJ32 (5 μM)	0.0162 ± 0.026	(0.73)	0.0582 ± 0.051	(2.59) **
+Verapamil (5 μM)	0.0179 ± 0.0043	(0.80)	0.0415 ± 0.0055	(1.85) **

^a^ IC_50_ values are represented as mean ± SD of at least three independent experiments performed in triplicate; ^b^ Values represent fold-of-resistance (FR) obtained by dividing IC_50_ value of antineoplastic drug in HEK293/pcDNA3.1 and HEK/ABCB1 cells with or without reversal agent divided by the IC_50_ value of respective antineoplastic drug in HEK293/pcDNA3.1 cells without reversal agent. Cell survival assay was determined by the MTT assay as described in Section 2. Verapamil was used as a positive control of P-gp inhibitor; * *p* < 0.05; ** *p* < 0.01 *versus* the control group without reversal agent.

Drug selected SW620/Ad300 cells may have multiple drug resistant mechanisms besides P-gp-mediated action. Therefore, the effect of sipholane analogs on P-gp was studied in HEK293 cells transfected with the *ABCB1* gene expression vector or empty pcDNA3.1 vector. The transfected HEK/ABCB1 cell was developed to solely exhibit P-gp-mediated resistance. In this cell line, the resistant folds were more significantly reduced by verapamil, SSJ26, and SSJ32 as shown in Table 3. These findings further support the hypothesis that SSJ26 and SSJ32 mainly affect P-gp.

Similarly, cell survival curves were made by plotting the survival fraction of aforementioned cell lines at various paclitaxel concentrations. As shown in Figure 1B, in the first curve, the application of SSJ26 did not affect potency of paclitaxel, therefore SW620 (drug-sensitive) alone and SW620 with SSJ26 are visualized as almost identical curves. In contrast, SW620/Ad300 (drug-resistant) cells are more viable in the absence of SSJ26 but once they are treated with SSJ26, the kill curve shifts to the left and closer to that of parental SW620 cells. This demonstrates the proportional reversal trend of SSJ26 in inhibiting P-gp-mediated resistance with differing concentrations of paclitaxel. Similar reversal trends were seen with SSJ32 (Figure 1C) and with verapamil (Figure 1D).

In HEK293 cells transfected with the empty vector pcDNA3.1, no change in cell viability was seen when treated with differing paclitaxel concentrations alone or in combination with SSJ26, SSJ32, or verapamil (Figure 1E). In contrast, when HEK293 cells transfected with ABCB1were treated with SSJ26, SSJ32 or verapamil, the curves shifted to the left (Figure 1F). This demonstrated that P-gp overexpressing HEK/ABCB1 cells were re-sensitized to paclitaxel by SSJ26 and SSJ32 treatment

### 3.3. Effect of SSJ26 and SSJ32 on Transfected HEK/ABCC1 and HEK/ABCG2 Cells

HEK293 cells transfected with ABC transporter genes *ABCC1* and *ABCG2* were used for the experiments to evaluate if the SSJ-26 and SSJ-32 is only specifically affected to P-gp. Vincristine is a substrate of the MRP1/ABCC1. As shown in Table 4, application of SSJ26 or SSJ32 with vincristine did not result in a change in resistant fold for the HEK293/*ABCC1* cells. Similarly, the effect of SSJ26 and SSJ32 in ABCG2-transfected HEK293 cells was also tested with mitoxantrone, a known substrate of BCRP/ABCG2. Our results indicated that SSJ26 and SSJ32 did not show reversal effect on *ABCG2*-mediated MDR. It can be concluded, thus far, that SSJ26 and SSJ32 are potent reversal agents of P-gp-mediated MDR but not of MRP1/ABCC1 or BCRP/ABCG2.

**Table 4:** Effects of SSJ26 and SSJ32 on the MRP1/ABCC1- and BCRP/ABCG2-mediated multidrug resistance.

Treatment	HEK293/pcDNA3.1		HEK293/ABCC1	
	IC_50_ ± SD ^a^ (μM)	FR ^b^	IC_50_ ± SD ^a^ (μM)	FR ^b^
Vincristine	0.0208 ± 0.0021	(1.00)	0.4731 ± 0.0282	(22.75)
+SSJ26 (5 μM)	0.0193 ± 0.0019	(0.93)	0.4693 ± 0.0184	(22.56)
+SSJ32 (5 μM)	0.0235 ± 0.0027	(1.13)	0.4897 ± 0.0639	(23.54)
+PAK-104P ^c^ (5 μM)	0.0189 ± 0.0023	(0.91)	0.0778 ± 0.0092	(3.74) **
**Treatment**	**HEK293/pcDNA3.1**		**HEK293/ABCG2**	
	**IC_50_ ± SD ^a^ (μM)**	**FR ^b^**	**IC_50_ ± SD ^a^ (μM)**	**FR ^b^**
Mitoxantrone	0.0309 ± 0.0023	(1.0)	0.4259 ± 0.0203	(13.8)
+SSJ26 (5 μM)	0.0294 ± 0.0019	(0.95)	0.3729 ± 0.0553	(12.0)
+SSJ32 (5 μM)	0.0333 ± 0.0028	(1.08)	0.3654 ± 0.0675	(11.8)
+FTC ^d^ (5 μM)	0.0273 ± 0.0032	(0.88)	0.0381 ± 0.0435	(1.2) **

^a^ IC_50_ values are represented as mean ± SD of at least three independent experiments performed in triplicate; ^b^ Values represent the fold-of-resistance (FR) obtained by dividing IC_50_ value of antineoplastic drug in HEK293/pcDNA3.1, HEK/ABCC1 and HEK/ABCG2 cells with or without reversal agent divided by the IC_50_ value of respective antineoplastic drug in HEK293/pcDNA3.1 cells without reversal agent. Cell survival assay was determined by the MTT assay as described in Section 2. PAK-104P was used as a positive control of ABCC1 inhibitor. FTC was used as a positive inhibitor of ABCG2; ^c^ PAK-104P: a pyridine analogue, 2-(4-(diphenylmethyl)-1-piperazinyl)ethyl-5-(trans-4,6-dimethyl-1,3,2-dioxaphosphorinan-2-yl)-2,6-dimethyl-4-(3-nitrophenyl)-3-pyridinecarboxylate P-oxide. Positive control of ABCC1 reversal agent; ^d^ FTC: The fungal toxin fumitremorgin C. Positive control of ABCG2 reversal agent; * *p* < 0.05; ** *p* < 0.01 *versus* the control group without reversal agent.

### 3.4. SSJ26 and SSJ32 Increased Intracellular Accumulation of [^3^H]-Paclitaxel by Inhibiting Its Efflux

**Figure 2:**
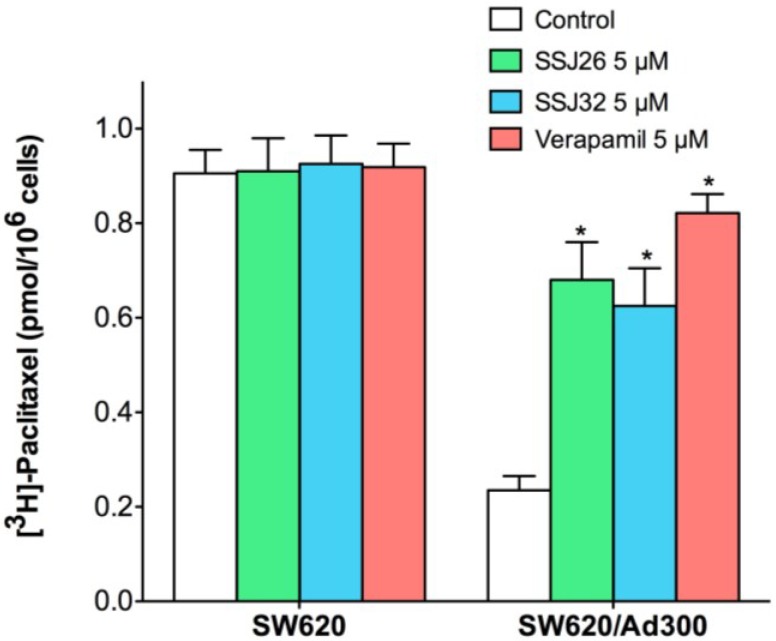
Effects of SSJ26 and SSJ32 on intracellular accumulation of [^3^*H*]-paclitaxel. The accumulation of [^3^*H*]-paclitaxel was measured after the cells were pre-incubated with or without SSJ26, SSJ32 or verapamil for 2 h at 37 °C and then incubated with 0.01 μM [^3^*H*]-paclitaxel for another 2 h at 37 °C. Columns are the mean of triplicate determinations; error bars represent SD. * Indicates *p* < 0.05 *versus* the control group.

To further elucidate the mechanism of drug resistance reversal, a drug accumulation study was performed. The results shown in Figure 2 demonstrated that intracellular paclitaxel concentration in SW620 treated with SSJ26, SSJ32, or verapamil did not differ significantly. However, the level of intracellular paclitaxel was much lower in SW620/Ad300 cells as compared with that in parental SW620 cells. When SSJ26, SSJ32, or verapamil was added, the intracellular levels of paclitaxel in SW620/Ad300 increased to almost comparable to that of SW620 cells. This demonstrates that the increased potency of paclitaxel is a result from increased intracellular accumulation when combined with SSJ26, SSJ32 or verapamil.

**Figure 3:**
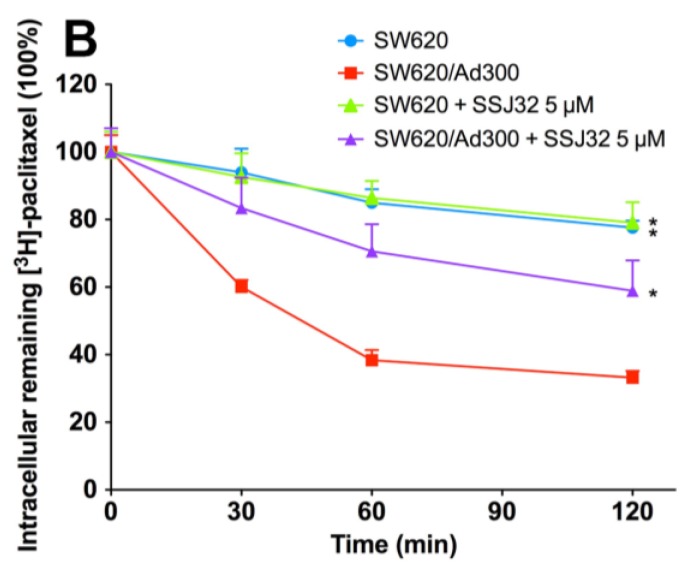
Effects of SSJ26 and SSJ32 on efflux of [^3^*H*]-paclitaxel. (**A**) The effect of SSJ26 on the efflux of [^3^*H*]-paclitaxel from SW620 and SW620/Ad300 cells; (**B**) The effect of SSJ32 on the efflux of [^3^*H*]-paclitaxel from SW620 and SW620/Ad300 cells. A time course *versus* percentage of intracellular [^3^*H*]-paclitaxel remaining (%) was plotted (0, 30, 60, 120 min). Lines are the mean of triplicate determinations; error bars represent SD. * Indicates *p* < 0.05 *versus* the control group.

Then, we conducted efflux assay to further explore the mechanism of intracellular [^3^*H*]-paclitaxel accumulation. In this experiment, intracellular [^3^*H*]-paclitaxel levels were measured at various time points after removal of extracellular [^3^*H*]-paclitaxel. At different time points, cells were harvested and the radioactivity was measured. As shown in Figure 3A, while the paclitaxel level significantly decreased in SW620/Ad300 drug-resistant cells, the levels of paclitaxel remained stable in parental SW620 cells. After treatment with SSJ26, the intracellular remaining [^3^*H*]-paclitaxel was significantly increased at all time-points. The treatment of SSJ26 did not alter the [^3^*H*]-paclitaxel levels in SW620 cells. Similarly, SSJ32 treatment also inhibited the efflux of [^3^*H*]-paclitaxel in SW620/Ad300 (Figure 3B). However, SSJ32 is not as potent as SSJ26 in inhibiting efflux of [^3^*H*]-paclitaxel. 

### 3.5. SSJ26 and SSJ32 Stimulated ATPase Activities but Did not Alter the Expression Level of P-GP

**Figure 4:**
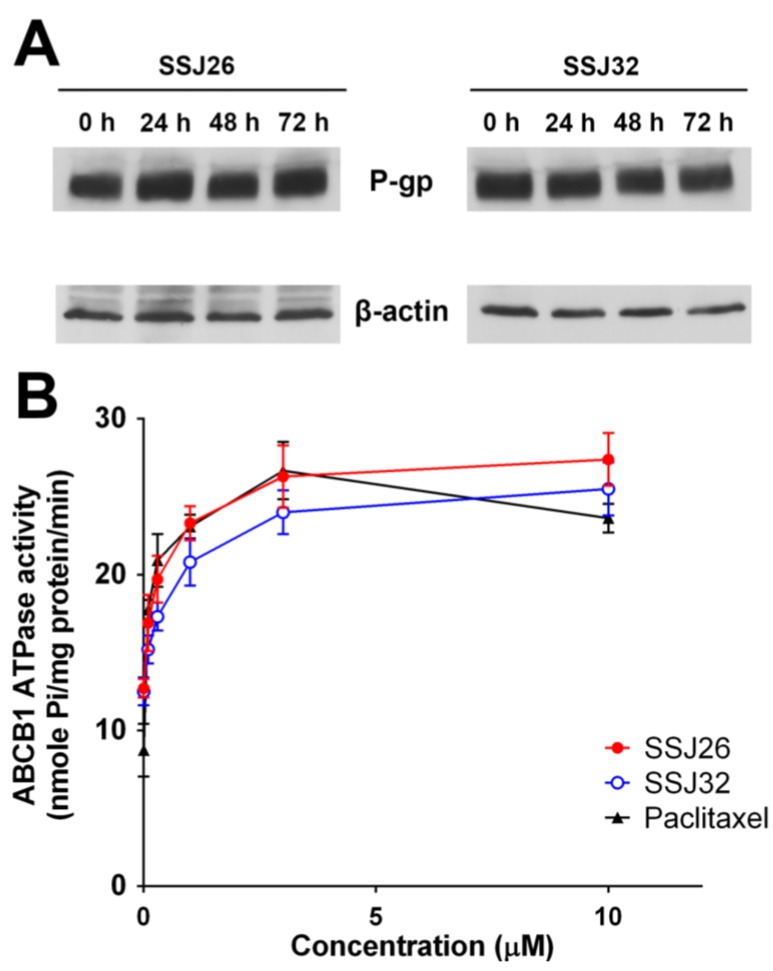
Effects of SSJ26 and SSJ32 on ATPase activity and expression level of P-gp. (**A**) Effect of SSJ26 at 5 μM and SSJ32 at 5 μM on expression level of P-gp in SW620/Ad300 cells for 0, 24, 48 and 72 h; (**B**) The effect of increasing concentration (0 to 10 μM) of SSJ26 and SSJ32 on the Vi-sensitive P-gp ATPase activity.

In order to determine whether the sipholenol analogs reverse drug resistance by affecting the P-gp expression, Western blot analysis was performed. As shown in Figure 4A, P-gp expression remains comparable both in presence and absence of treatment with SSJ26 and SSJ32 at 5 μM up to 72 h. Since these compounds do not alter protein expression, the reversal effect is probably due to inhibition of P-gp transporter function. P-gp/ABCB1 transporter utilizes energy derived from the hydrolysis of ATP to efflux its substrates across the membrane against a concentration gradient, thus ATP consumption reflects its ATPase activity. To assess whether SSJ26 and SSJ32 have any effect on the ATPase activity of P-gp/ABCB1, we measured P-gp/ABCB1-mediated ATP hydrolysis in the presence of SSJ26 or SSJ32 at various concentrations from 0 to 10 μM. Interestingly, SSJ26 stimulated the ATPase activity of P-gp/ABCB1 in a concentration-dependent manner, with a maximal stimulation of 2.15-fold of the basal activity. In contrast, the maximal stimulation of SSJ32 was 2.03-fold of the basal activity. The Figure 4B demonstrates that the concentration of SSJ26 required to obtain 50% stimulation is 0.26 μM. The Figure 4B also demonstrates that the concentration of SSJ32 required to obtain 50% stimulation is 0.60 μM.

### 3.6. Binding Mode of SSJ26 and SSJ32 with Homology Model of P-GP

In the absence of the crystal structure of human P-gp, we developed a homology model, based on the refined crystal structure of mice P-gp. To understand the molecular interactions of SSJ26 (Sipholenol A-4-*O*-acetate) and SSJ32 (Sipholenol A-4-*O*-isonicotinate) with human P-gp, docking simulations were performed at pre-generated grid (Section 2). The docking scores were found to be −7.894 kcal/mol for SSJ26 and −6.956 kcal/mol for SSJ32, respectively.

**Figure 5:**
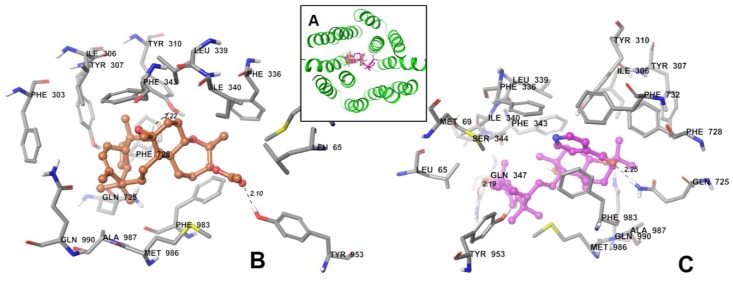
XP-Glide predicted binding mode of SSJ26 and SSJ32 with homology modeled P-gp. (**A**) Docked poses of SSJ26 and SSJ32 into drug binding sites of human P-gp. Backbone of human P-gp is depicted as gray ribbons viewed from the intracellular side of the protein looking into the internal chamber. SSJ26 and SSJ32 are represented as tube models with the same color scheme below; (**B**) Binding mode of SSJ26 within human P-gp. Important residues are depicted as tubes with the atoms colored as carbon—gray, hydrogen—white, nitrogen—blue, oxygen—red, sulfur—yellow, whereas SSJ26 is shown as ball and stick model with the same color scheme as above except carbon atoms are represented in orange. Dotted black lines indicate hydrogen bonds; (**C**) Binding mode of SSJ32 within the human P-gp. The color scheme is same except carbon atoms are represented in purple.

The docked poses of SSJ26 and SSJ32 into the large drug-binding cavity of human P-gp were shown in Figure 5A. The docked poses of the two ligands were found to be overlapped, however in different molecular orientations. The docked pose of SSJ26 is shown in Figure 5B. The sipholenol A core structure was mainly stabilized into a large hydrophobic pocket formed by several hydrophobic and aromatic residues, such as Leu65, Met69, Phe303, Ile306, Tyr307, Tyr310, Phe336, Leu339, Ile340, Phe343, Gln725, Phe728, Tyr953, Phe 983, Met986, Ala987, and Gln990. The C-4-*O*-acetate substituent on the hexahydro-oxepine ring was involved in hydrogen bonding interaction with Try925 (-CO···HO-Tyr953, 2.10 Å), suggesting the importance of the ester substituent. Also, the C_19_-hydroxyl group of the hexahydroazulen ring interacted with Try310 through hydrogen bonding contact (-OH···HO-Tyr310, 2.22 Å). 

The docked pose of SSJ32 was found to be rotated as a result of the replacement of the acetate of SSJ26 with a heteroaromatic ester isonicotinate in this compound as shown in Figure 5C. The pyridine ring may be stabilized by nearby aromatic residues Phe336, Phe732 and Phe983. SSJ32 may form two hydrogen bonding interaction with Gln347 (OH···HO-Gln347, 2.19 Å) and Gln725 (CO···H_2_N-Gln725, 2.25 Å). However, the change of substituent in SSJ32 seems to bring less tight interactions (255 good contacts counts as compared to 386 in SSJ26) and may lead to imperfect fit with the hydrophobic pocket. This may explain its relatively poorer reversal activity as well as Glide docking score. 

## 4. Discussion 

As cancer continuously evolves, there are powerful antineoplastic therapy regimens that are being developed exponentially. The compounds investigated in this study could contribute to these treatments as significant novel chemotherapy enhancement. P-gp is considered as a strong therapeutic target for MDR reversal agents. A number of natural compounds extracted from marine sponges and their semisynthetic derivatives have previously demonstrated effectiveness against multi-drug resistance in cancer cells [16]. In this study, esters of the marine natural triterpene sipholenol A, SSJ26 and SSJ32, have shown practical abilities to reverse P-gp-mediated drug resistance and their reversal mechanisms were elucidated. SSJ26 and SSJ32 can re-sensitize cells that have developed drug-resistance to chemotherapeutic agents that are substrates of P-gp such as doxorubicin and paclitaxel. They stabilized the level of intracellular [^3^H]-paclitaxel accumulated in the cells by inhibiting P-gp mediated drug efflux in a competitive manner. The expression of P-gp protein was not compromised in presence or absence of these reversal agents.

In this study, MTT assay was used to determine the IC_50_ values to quantify the sensitivity of cells to the experimental sipholenol analogs. These compounds were first tested on human colonic adenocarcinoma cells SW620 to visualize their effects on the cancer cells. All the tested compounds have no direct cytotoxic effect on the cells, indicating that they might act through enhancing chemotherapeutic activity of anti-neoplastic agents. These sipholenol analogs went through a further screening and the compounds that have the most potent MDR reversal effect were identified to be SSJ26 and SSJ32. 

Various concentrations of SSJ26 and SSJ32 were tested in combination with two chemotherapy agents, doxorubicin and paclitaxel, which are known substrates of P-gp. At increasing concentrations of 1.25, 2.5, and 5 μM of each of the compounds, the FR proportionally decreased, suggesting concentration dependent P-gp inhibition. The FR values at 5 μM were similar to those of verapamil. Cisplatin was used as a negative control drug, since it does not submit to the multi-drug resistance effect of P-gp. Theoretically then the IC_50_ values of cisplatin on SW620 and SW620/Ad300 should remain the same. As predicted, the IC_50_ values of cisplatin were similar in both parental and drug resistant cells, and the application of SSJ26, SSJ32, or verapamil did not significantly alter the resistance fold as well. To further clarify their specificity, these compounds were tested in *ABCB1*-transfected HEK293 cell lines. The transfected cells exhibit resistance of a single mechanism, while the drug-selected cells previously mentioned have resistance induced by doxorubicin and a variety of diverse unidentified mechanisms may be present. At the same concentration of 5 μM, the decrease in drug resistance was significantly greater in HEK/ABCB1 than in SW620/Ad300, indicating the sipholanes may specifically target *ABCB1* mediated MDR. Their sole effect on *ABCB1*-mediated resistance was bolstered by treating HEK293 cells transfected with other known MDR transporter expression vectors, specifically *ABCC1* and *ABCG2*. Vincristine is a known substrate of transporter proteins coded by *ABCC1*, and mitoxantrone, of *ABCG2*. Overexpression of ABCC1 or ABCG2 confers resistance to the aforementioned antineoplastics, which can be significantly reversed by PAK104P or FTC, respectively. When compared to these MDR inhibitors, SSJ26 and SSJ32 negligibly decreased the resistance of *ABCC1-* and *ABCG2*-transfected cells. This evidently indicated how precise the affinity of these compounds is to *ABCB1*-expressing P-gp.

The mechanism of reversal effect of sipholenol triterpenoids is mainly by the inhibition of the P-gp efflux activity. This can be supported by the [^3^*H*]-paclitaxel intracellular accumulation and the efflux study. Our results show that while the amount of intracellular paclitaxel was significantly lower in the drug resistant SW620/Ad300 cells, the accumulation of paclitaxel increases with the addition of SSJ26, SSJ32, or verapamil. As time increases, drug efflux transporter P-gp removes an increasing amount of drug from SW620/Ad300 cells. This efflux rate decreases significantly in response to SSJ26 and SSJ32 when treated in combination with paclitaxel. 

Immunoblotting experiment showed that SSJ26 and SSJ32 did not change the level of P-gpexpression in cells, insinuating that these compounds impact on the function of P-gp protein. Interestingly, SSJ26 stimulated the ATPase activity of P-gp in a concentration-dependent manner, with a maximal stimulation of 2.15-fold of the basal activity. On the other hand, the maximal stimulation of SSJ32 is 2.03-fold of the basal activity. The concentration of SSJ26 and SSJ32 required to obtain 50% stimulation is 0.26 μM and 0.60 μM, respectively. It can be concluded that SSJ26 had a more potent effect on the ATPase activity of P-gp than SSJ32, which was consistent with other results. Furthermore, as shown in Figure 4B, SSJ26 and SSJ32 interacted with P-gp transporter in a similar manner to paclitaxel, indicating that these two compounds might be the substrates of P-gp transporter. Therefore, SSJ26 and SSJ32 may be actively transported out by P-gp, competitively inhibit P-gp mediated efflux of other substrates. Abraham *et al.* reported that Vi-sensitive P-gp ATPase activity was stimulated by sipholenone E, sipholenol L, and siphonellinol D in a concentration-dependent manner with maximum stimulation of over 2.1-, 1.7- or 1.5-fold, respectively. The apparent Km values for sipholenone E, sipholenol L or siphonellinol D were ~14 μM, 5 μM and 4 μM, respectively [30]. Interestingly, the Km values for SSJ26 and SSJ32 were 0.023 μM and 0.07 μM, respectively. Therefore, SSJ26 and SSJ32 had a more potent effect on the ATPase activity of P-gp than sipholenone E, sipholneol L or siphonellinol D. This statement can be further visualized by molecular modeling studies. *In silico* molecular orientations of SSJ26 and SSJ32 at the P-gp homology model suggest varying binding affinities and justify their difference in potency as MDR-reversal agents. The Glide docking score estimates the ligand binding’s free energy between the substrate and its binding site [31]. In cell-based assays, SSJ26 showed stronger potency than SSJ32 in modulating P-gp-mediated MDR. The binding energy scores expressed in kcal/mol for the sipholenol A acetate SSJ26 against human P-gp was found to be −7.894. Relatively lower binding energy score was noticed for the isonictoninate ester SSJ32 against ABCB1 (−6.956), thus strengthening the applicability and relevance of human P-gp homology model for virtual studies of binding modes. SSJ32, based on its reversal effect, is a weaker inhibitor of P-gp than SSJ26, which could partially explain its poor binding energy score [32]. Though docking is a useful tool to understand ligand-protein interactions, the present study involves P-gp, which are particularly challenging because it possesses a large drug-binding cavity. Currently, no high-resolution crystal structure of human P-gp is available and this prompted the use of homology model. Without co-crystal complexes with P-gp, docking using P-gp homology model can be used for preliminary assessment of binding modes and affinities of large number of unknown hits.

## 5. Conclusions

In conclusion, this study demonstrated the potential of the marine natural triterpene sipholenol A-4-*O*-esters as P-gp mediated MDR reversal agents. Their strong affinity and specificity to *ABCB1* expressing efflux proteins would decrease the possibility of other pharmacokinetic interactions with pharmacological agents. Future research will propel these invaluable compounds to be paired with chemotherapeutic drugs in order to deliver advancement in cancer patient care. 

## Supplementary Files

Supplementary File 1
