# Supplementary material for: Esters of the Marine-Derived Triterpene Sipholenol A Reverse P-GP-Mediated Drug Resistance
Source: Mar Drugs. 2015 Apr 14;13(4):2267–86. doi: 10.3390/md13042267 (PMC4413211; doi:10.3390/md13042267)
Supplement: Supplementary File 1 [file marinedrugs-13-02267-s001.pdf]

Supplementary Information

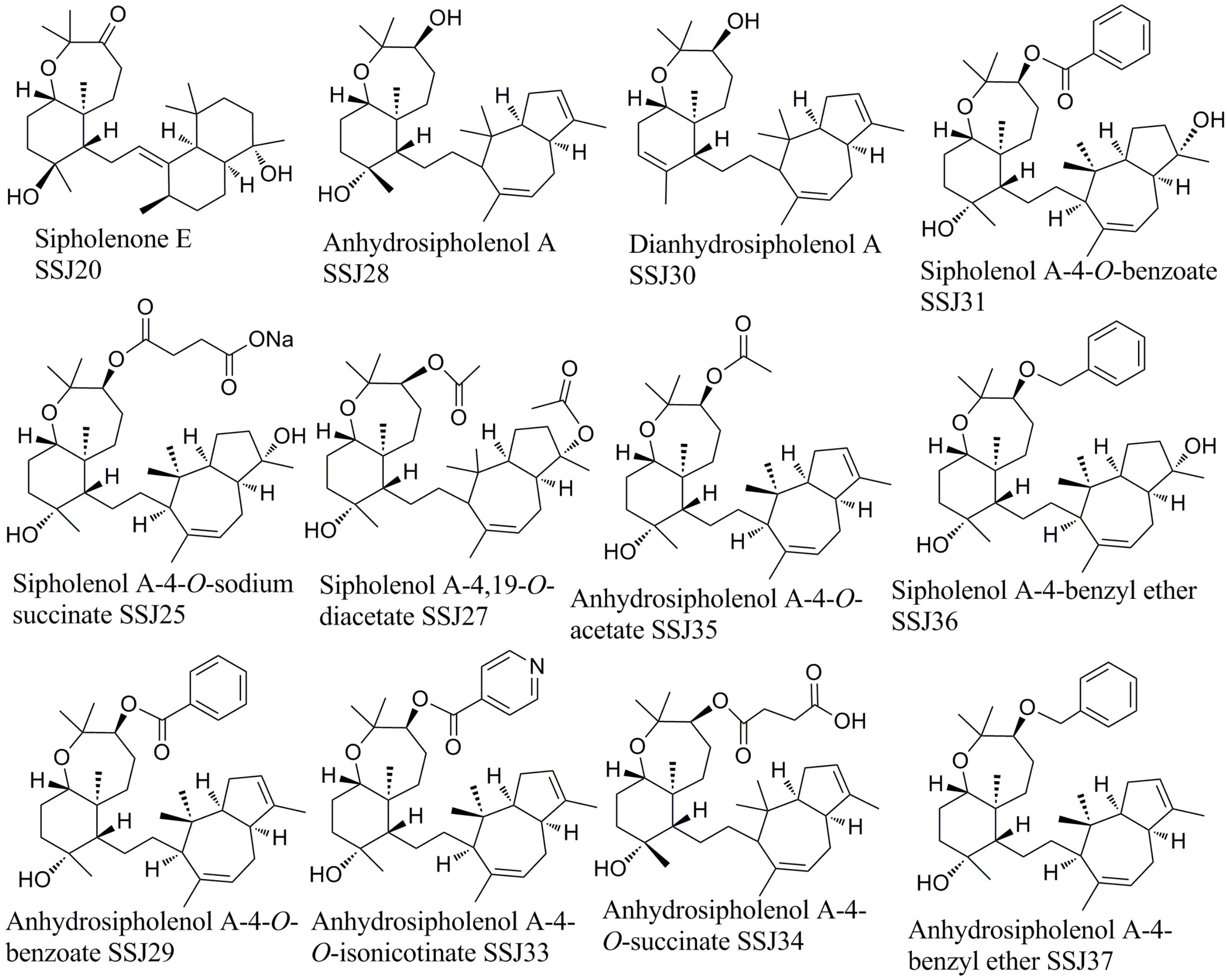

Figure S1. Structures of tested sipholane analogs.

Table S1. Cytotoxicity of 14 SSJ compounds against SW620 and P-gp over-expressing SW620/Ad300 cells [a].

**Compounds**	**IC_{50} (μM) [b]**	
	**SW620**	**SW620/Ad300**
SSJ20 (1)	>30.0	>30.0
SSJ25 (2)	>30.0	>30.0
SSJ26 (3)	>30.0	>30.0
SSJ27 (4)	>30.0	>30.0
SSJ28 (5)	>30.0	>30.0
SSJ29 (6)	>30.0	>30.0
SSJ30 (7)	>30.0	>30.0
SSJ31 (8)	>30.0	>30.0
SSJ32 (9)	>30.0	>30.0
SSJ33 (10)	>30.0	>30.0
SSJ34 (11)	>30.0	>30.0
SSJ35 (12)	>30.0	>30.0
SSJ36 (13)	>30.0	>30.0
SSJ37 (14)	>30.0	>30.0

[a] Cell survival was determined by MTT assay as described in Section 2.3; [b] IC_{50} values are represented from at least three independent experiments performed in triplicate.

© 2015 by the authors; licensee MDPI, Basel, Switzerland. This article is an open access article distributed under the terms and conditions of the Creative Commons Attribution license (http://creativecommons.org/licenses/by/4.0/).
